# Supplementary material for: Content-rich biological network constructed by mining PubMed abstracts
Source: BMC Bioinformatics. 2004 Oct 8;5:147. doi: 10.1186/1471-2105-5-147 (PMC528731; doi:10.1186/1471-2105-5-147)
Supplement: Additional File 2 — The original results of the above study (non-essential files are deleted to keep the file size under the limit set by BMC bioinformatics). [file 1471-2105-5-147-S2.bz2 › chilibotAdditionalFile2/dip20/53ID8144619E207/html/BW_W.html]

 


 **BW** and **W** 
  
Found 427 abstracts in PubMed, retrieved 20.  
 

 What does Google say? 
 PDF only 
| .edu only 

---

**Interactive relationship** (e.g. stimulation, inhibition, etc)

**Neutral relationship**- In these equations,  **W**  is  **BW**  kg, T is temperature degrees C, and WG is daily weight gain g.  Ref: 12705403 Poult Sci, 2003
- 50 and 00 ppm and diazinon 1 0 and 90 mg k  **bw**  were administered to rats via gavage PCBs and diazinon or via drinking water Pb daily for 5  **w** .  Ref: 12882489 Vet Hum Toxicol, 2003

**Non-interactive relationship** (e.g. studied together, co-existance, homology, etc.)

- , Brown  [ **BW** ] ,  **W** .  Ref: 12765548 Biochem J< MedlineTA>Biochem J, 2003
- The objective of this study was to evaluate the protective role of isoflavones 2 mg kg B. **W**  on semen quality and plasma testosterone levels of male New Zealand White rabbits given sublethal dose 24 mg kg  **BW**  every other day for 12 weeks of cypermethrin.  Ref: 12856928 J Environ Sci Health B, 2003
- Compared with estimated high background reference concentrations of 10 ng TEQ PCDF PCDD kg  **w** . **w** . in liver of cod and in hepatopancreas brown  [ **BW** ]  meat of crabs, Frierfjord samples in 2001 were about 60 and 70 times higher, respectively.  Ref: 12738288 Chemosphere< MedlineTA>Chemosphere, 2003
- The IC50, minimum inhibitory concentration MIC, minimum microbicidal concentration MMC and minimum microbistatic concentration MMS varied considerably depending on the microorganism tested, the sensitivity decreasing as follows S. aureus > P. aeruginosa S sensitive > E. coli S > P. aeruginosa R resistant > E. coli R > B. subtilis > Z. ramigera > C. albicans > S. cerevisiae > A. pullulans B black > A. pullulans  **W**  white > T. viride Br brown  [ **BW** ]  > M. gypseum > A. niger > F. nivale > P. chrysogenum > T. viride G green.  Ref: 12422513 Folia Microbiol Praha , 2002
- **W**  kg  **bw** , or relative maximal oxygen consumption VO 2 max.  Ref: 12205584 J Magn Reson Imaging, 2002
